# Analysis of factors delaying the surgical treatment of patients with neurological deficits in the course of spinal metastatic disease

**DOI:** 10.1186/s12904-018-0295-3

**Published:** 2018-03-07

**Authors:** Grzegorz Guzik

**Affiliations:** Orthopedic Oncology Department, Specialist Hospital in Brzozów- Podkarpacki Oncology Center, ul. Dworska 77a, 38-420 Korczyna, Polska Poland

**Keywords:** Metastases, Spinal tumors, Surgical treatment of the spine, Resections of spinal tumors, Spinal stabilization, Neurological deficits, Frankel scale

## Abstract

**Background:**

Thoracic spine cancer metastases is frequently the cause of neurological deficits. Despite the availability of diagnostics, delays in treatment are still quite common. The aim of this work is to analyze the reasons for delayed diagnostics and treatment, in patients with neurological deficits in the course of metastatic spine disease.

**Methods:**

In our study patients medical data was analyzed from 2013 to 2015. The analysis covered the following aspects: symptoms of metastases, time of neurological deficits occurrence, where and when initial diagnostics were performed, time from diagnosis to proper surgical treatment in an oncological centre. In total, 411 patients were consulted and 287 were operated on. Of 112 patients with neurological deficits, 64 underwent surgeries. Women represented the majority of the patients. The most common primary neoplasms were breast cancer and myeloma.

**Results:**

In 75% of the patients neurological symptoms occurred prior to admission to a hospital. The average time between the onset of neurological symptoms and medical consultation was 4 days. The patients were diagnosed mainly at neurologic, orthopedic and emergency departments. The mean time between undergoing radiological examinations and receiving the examinations results was 2.4 days for CT and 2.8 days for MRI. The average time between a patients’ admission from the department where they were initially diagnosed, to the orthopedic oncology ward was 4.5 days.

**Conclusions:**

The most common cause of the delayed treatment of patients with neurological deficits, in the course of metastatic spine disease, is a combination of the lack of knowledge among patients and healthcare personnel regarding the necessity of early diagnosis.

## Background

The most common location for cancer metastases is the spine. The most frequently affected areas are the thoracic spine (70%) followed by the lumbar spine (20%), and lastly the cervical spine (10%). 70% of thoracic metastases are associated with clear clinical symptoms and 5–15% develop neurological deficits. Pain associated with lumbar spine involvement affects 21.6% of patients; in the cervical spine 8.1% [1–3].

The symptoms of spinal metastases are non- specific and may occur in the course of other spine diseases. Diagnostics is often delayed, making effective treatment difficult or impossible. The symptoms preceding paralysis or paresis include: intensive pain, change in its characteristics, non-characteristic sensations (tingling, feeling of warmth or cold, muscular tremor, feeling of heavy legs), increased pain during coughing and defecating, and reduced mobility. The occurrence of these symptoms should urge the patient and his/her family, friends and doctors to conduct early diagnostics, including CT and MR imaging. An early and thorough neurological assessment is also of importance [1, 4–7].

The aim of this work was to analyze the reasons for delayed diagnostics and treatment of spine metastases causing neurological deficits.

## Methods

In our study, patients data was analyzed from the years 2013–2014.

The time between the occurrence of neurological deficits and medical consultation was analyzed. The timeframe from the patients initial diagnosis, admission to the hospital and transfer to the proper spinal ward in an oncological centre, was analyzed. Contra-indications to surgical treatment were considered.

The inclusion criteria for the study was the occurrence of peripheral nervous system dysfunction in the course of histhopatologically confirmed metastatic disease of the spine. Undiagnosed patients, and patients with other diseases (tuberculosis, inflammation, primary neoplasm of the spine), were excluded from the study.

**Fig. 1:**
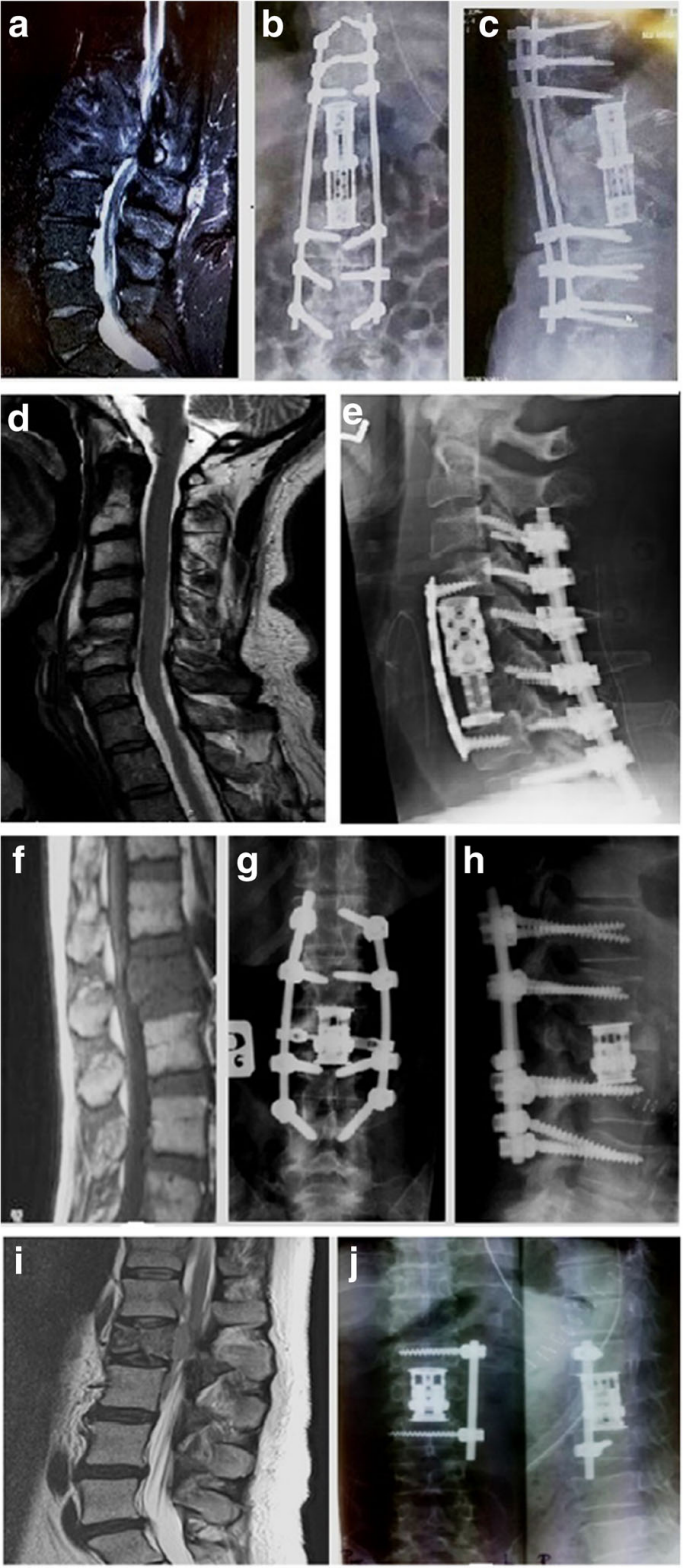
MRI image of metastatic thyroid cancer located in thoracic 11–12 and lumbar 1 vertebrae (**a**). Postoperative radiograms (**b**, **c**) showing vertebral prosthesis and posterior spine fixation. MRI images of C5-C6 breast cancer metastasis (**d**), and after tumor resection and cervical spine reconstruction (**e**). MRI image of metastatic breast cancer in the second lumbar vertebrae (**f**). Postoperative radiograms (**g**, **h**) after 360 degree reconstruction of the spinal column. MRI of fractured second lumbar vertebrae (**i**) and after prosthesis implantation and lateral spinal fixation (**j**)

In total, 411 patients were consulted in our department, of which 344 were hospitalized and 287 were surgically treated. Of 411 patients, 112 of these presented neurological deficits. Neurological deficits were noted in 68 of 344 patients hospitalized in our ward and in 64 of 287 patients treated surgically. Figure 1 demonstrate examples of metastatic tumors of the spine, causing pathological fractures and spinal cord damage.

Women represented the majority of the patients, accounting for 255 cases (62%). The median age was 63 years for women, and 68 years for men. The primary malignancies were cancer of the breast (127), myeloma (89), kidney (44), prostate (42), lymphoma (24), lung (21), colon (16), thyroid (11), gastric (8), and others (29) (Table 1).

**Table 1:** Number of patients with metastases to the spine and neurological deficits in relation to the type of neoplasm

Type of neoplasm	Total number of patients with metastases (411)	Number of patients with neurological deficits (112)
Breast cancer	127	38
Myeloma	89	26
Kidney cancer	44	12
Prostate cancer	42	13
Lymphoma	24	6
Lung cancer	21	4
Colon cancer	16	2
Thyroid cancer	11	2
Gastric cancer	8	2
Others	29	7

The average time from the primary cancers diagnosis to spine metastases diagnosis was 11 months (range 2–54 months). Table 2 exhibits the mean duration from the diagnosis of metastatic disease, to the onset of neurological deficits.

**Table 2:** The mean duration time from spine metastases discovery to neurological deficits occurrence

Type of neoplasm	The mean duration time from metastatic spine disease diagnosis to neurological deficits occurrence (months)
Breast cancer	26
Myeloma	5
Kidney cancer	14
Prostate cancer	31
Lymphoma	7
Lung cancer	3
Colon cancer	21
Thyroid cancer	31
Gastric cancer	3
Others	6

Neurological assessment included the examination of muscle strength, sensation and tendon reflexes. The strength of muscles was evaluated using the Lovett scale (0- paralysis, 5- normal). Sensory examination included pain, touch, warm and cold feeling (0- loss of sensation, 2- normal sensation). Tendon reflexes examination evaluated the effectiveness of both the upper and lower limbs (0- absent reflex, 1- seen with reinforcement, 2- normal, 3- very brisk, 4- clonus). Severity of neurological deficits was assessed according to the Frankel classification (A-complete impairment, E- normal). The findings of these examinations are presented in Table 3.

**Table 3:** Severity of neurological deterioration in relation to the type of primary neoplasm

Type of neoplasm	Frankel A	Frankel B	Frankel C	Frankel D
Breast cancer	9	13	10	6
Myeloma	6	9	8	3
Kidney cancer	3	6	2	1
Prostate cancer	4	6	1	2
Lymphoma	3	2	–	1
Lung cancer	1	–	1	2
Colon cancer	–	–	2	–
Thyroid cancer	–	–	1	1
Gastric cancer	–	1	–	1
Others	1	5	1	–

Of 411 patients, 124 were disqualified from surgical treatment due to the following reasons: bad general condition (76 patients), no consent to surgical intervention (20 patients), tumor extensions (large multi-segmental lesions, extensive vascularisation, skin lesions at the surgical site) - (18 patients), age (10 patients). In a group of 112 patients with neurological deficits, 24 patients were disqualified from the treatment, due to poor general health (16 patients) and long-lasting paralysis, accompanied by poor prognosis and morphological changes of the spinal cord (8 patients).

## Results

In the majority of the patients (84, 75%) neurological deficits prior to admission to the hospital were present. In 22 patients (20%), the symptoms were diagnosed during a routine medical examination in the outpatient oncology clinics in our Hospital. In 6 patients (5%) the onset of neurological deficits occurred suddenly, in the course of oncological treatment in our facility.

The patients who were conscious of the fact that their neurological status was worsening, visited their physicians after various periods of time. The average time for this delay was 4 days (range 1–16 days). In patients treated in the oncology clinics, the onset of sensory impairments and minor pareses was not established in the medical treatment records. Only 11 out of 84 patients with neurological deficits were directly referred to the emergency department in our hospital. Those were the patients who live in our city, who had previously undergone oncological treatment in our hospital. The remaining 73 patients were primarily hospitalized at neurological, internal medicine, oncology, hematology and orthopedic wards, or diagnosed at the emergency departments of other hospitals. Table 4 shows the number of patients hospitalized at different wards, the median hospitalization time, diagnostic investigations and the time from radiological examinations to their results.

**Table 4:** Data concerning the diagnostics of 73 patients with neurological symptoms in the course of metastatic disease of the spine, before consultation in our department

Hospital wards	Number of patients	Mean hospitalizationtime (days)	Number of performed CT examinations	Number of performed MRI examinations	Mean wait time for CT results (days)	Mean wait time for MRI results (days)
Internal medicine	9	4,4	6	5	2,7	3,3
Neurological	22	7,8	14	13	3,7	4,2
Oncology	4	4,5	1	1	2	3
Hematology	3	4,3	1	–	3	–
Radiotherapy	2	3,5	1	–	2	–
Surgery	2	3,5	2	–	2	–
Rehabilitation	1	5	1	–	3	–
Orthopedic	11	2,7	11	7	1	1
Emergency Department	19	–	19	5	–	–

Prior to being referred to the oncology center, the patients were treated symptomatically. 37 patients received analgesics drugs and steroid hormones (Dexaven). In 27 patients orthopedics corsets were recommended. Antithrombotic prevention was introduced in 32 patients.

The date of spinal radiotherapy was determined by the “onco-team” with the participation of the orthopedic surgeon, oncologist and radiation therapist. The average waiting time for CT results was 2.4 days, and 2.8 days for MRI results. The mean time between patients’ admission to the hospital and orthopedic consultation in the oncology center was 4.5 days (this data does not include the patients diagnosed and examined at the emergency department who were admitted to the hospital on the same day).

## Discussion

Symptoms of neurological deterioration in conjunction with spinal diseases are a common indication for urgent surgical intervention. Treatment outcomes are determined by the mechanism of injury and the extent of the nerve structures damaged. These patients should be urgently admitted to special hospital wards, and have the necessary diagnostic evaluations for adequate treatment.

In oncologic patients neurological symptoms worsen over a long period of time. Knowledge of spinal metastases symptoms, by the patients, their families and doctors, often plays a crucial role in early diagnosis and the reduction in the risk of complications [1, 8, 9].

The study has confirmed the observations of other authors who concluded that most patients present prodromal signs of deep neurological dysfunction such as: changes in the intensity and type of pain, numbness, tingling in the limbs, general weakness and reduced mobility [1, 2, 10–13].

Spinazze indicated that 5–14% of patients with metastases of the spine presented symptoms of nervous structure damage. 96% of the patients reported increased pain during coughing and defecating. The most commonly reported symptoms of spinal cord compression were: dysesthesia (51–80% of the patients), decreased muscle strength and feelings of weakness (40–64%), urethral sphincter dysfunction (40–64%) [14].

Early and proper diagnosis is one of the factors which plays a crucial role in determining treatment outcome/s. Despite the availability of MRI and CT, there are still many cases of deep, inveterate damage to the nervous system, in the course of undiagnosed or inadequately treated metastatic disease of the spine. The ESCC (Epidural Spinal Cord Compression) scale differentiates between four grades of spinal cord compression, through tumor. Grade 0 means bone-only location of tumor and, due to a risk of neurological deficits, does not require urgent surgical intervention. Grades 1,2,3 represent compression of structures such as dura mater, spinal cord, and the obstruction of CSF flow. Such images are indications for urgent consultation in a spinal surgery centre. As is commonly known, enhanced treatment outcomes are achieved in patients with minimally intense and short-term dysfunctions. These patients are much more likely to rapidly regain their physical functions [1, 2, 15, 16].

Schoeggl et al. found that 25% of patients with neurological deficits, during the course of metastatic disease of the spine, improved neurologically after adequate treatment. There was no postoperative improvement in patients with complete limb paralyses. The best outcomes were reported in patients with minor pareses (68% of cases improved). Impaired sphincter function was positively affected in 18% of patients. The authors highlighted the need for early diagnostics and urgent surgical intervention as the two most crucial factors in determining the outcome [17].

Wals, Gokashlan et al. reported decreased pain in 76–100% of surgically treated patients, and neurological improvement in 50–76% of cases. The authors observed significantly improved outcomes in patients with less severe neurological deficits, 81–95% of whom improved [18, 19].

Finkelstain proved that the risk of death is 19% higher in patients with neurological complications, than in patients with no deficits. He concluded that the occurrence of neurological deficits is one of the most significant negative prognostic factors [8].

An overestimation of the effectiveness of radiotherapy may be one of the reasons for delayed treatment. Neither oncologists, nor palliative care physicians, neurologists and internists cooperate with orthopedic or neurosurgery specialists once spinal metastasis is diagnosed, but refer the patients to radiotherapy clinics or wards [2].

Patchell reported that 85% of patients after surgical treatment and 57% of patients who had undergone radiotherapy treatment for spinal metastases, regained their ability to work. 62% of patients who underwent surgical intervention, regained their ability to walk. This is in contrast with only 19% of patients treated with radiotherapy [20].

A common assumption still exists that there are no indications for spinal surgery in oncology patients due to their expectedly short survival.

The median survival times of patients with spinal metastases, reported by Tokuhashi, were: thyroid cancer- 25.6 months, breast cancer- 18.6 months, prostate cancer- 17.9 months, kidney cancer- 9.8 months, lung cancer- 5.2 months [21].

Bilski proved that surgical treatment prolongs survival in patients with metastases to the spine. The patients remain under outpatient care, their condition and quality of life are better [4, 5].

Studies by Kwok revealed that 30% of patients live longer than a year despite pareses or paralyses of limbs in the course of metastatic disease of the spine [22].

It is now believed that all patients should be qualified for surgical treatment if their survival prognosis exceeds 3 months and their general condition allows it. Among many scales introduced to facilitate qualification, the systems by Tomita, Tokuhashi, Asdourian, Karnofsky, and Harrington are most frequently used. It is essential that the qualification is multidisciplinary and multi-factorial [1, 2, 23].

Our study clearly suggests that both patients and healthcare practitioners ultimately bear the responsibility for delays in adequate surgical treatment. New, significant signs of the disease are either ignored or suppressed by patients. This lack of reaction is often the result of insufficient knowledge of the disease and its related hazards. Nurses and doctors still have insufficient knowledge concerning spinal metastases symptoms. A common assumption is that the effective treatment of spine metastases is impossible, thus delaying proper radiological examination, which can lead to a worsening in prognosis.

## Conclusions

The lack of patient knowledge, regarding the need for diagnostics, as soon as neurological symptoms occur, is the main reason for delayed surgical treatment. Diagnostics of oncology patients is not conducted at competent wards and lasts far too long, reducing the patient’s chances for effective treatment. Education of patients and healthcare practitioners dealing with cancer can significantly facilitate early and adequate diagnostics, and thus improve outcomes.

## Abbreviations

CSF: Cerebrospinal fluid
CT: Computed tomography
ESCC: Epidural Spinal Cord Compression
MRI: Magnetic resonance imaging
